# The Effects of Denture Cleansing Solutions on the Retention of Attachments of Implant Supported Overdentures

**Published:** 2015-03

**Authors:** Reza Derafshi, Mina Mohaghegh, Maryam Saki, Anahita Safari, Mohammad Rabee Haghighi

**Affiliations:** 1 Dept. of Prosthodontics, School of Dentistry, Shiraz University of Medical Sciences, Shiraz, Iran;; 2 Postgraduate Student in Orthodontics, Orthodontic Research Centre, Student Research Committee, Dept. of Orthodontics, School of Dentistry, Shiraz University of Medical Sciences, Shiraz, Iran;; 3 Undergraduate Student, School of Dentistry, Shiraz University of Medical Sciences, Shiraz, Iran;

**Keywords:** Denture retention, Implant overdenture, O-ring attachment, Denture cleanser

## Abstract

**Statement of the Problem:**

Implant-retained overdenture can improve the stability of dentures and prevent bone loss. Overdenture-wearing patients need special hygiene care.

**Purpose:**

The aim of this study was to evaluate the effects of various denture cleansers on the retention of Dio orange O-rings.

**Method and Materials:**

In this experimental study, 40 Dio orange O-rings were divided into 4 groups (10 O-rings each) and each group was soaked for equivalent of 6 months in the following solutions: 5.25% NaOCl (1:10 dilution), Corega cleanser tabs, Professional cleanser tabs and water (as the control group). After 6 months, O-rings were tested for 2inch/minutes of tensile force. The peak load-to-dislodgement was recorded. Data were imported to SPSS18 and were analyzed by One-Way ANOVA and Tukey HSD test (*p*≤ 0.05).

**Results:**

Denture cleansing solutions have significant effects on the reduction of retentive value of O-rings (*p*≤ 0.001). Corega tabs caused the reduction of 15.7% (9.91±0.53 N) in the retentive value of O-rings and Professional tabs caused 15% (10.00±0.86 N). NaOCl caused significant decrease (48%) in retentive value of O-rings (6.10±0.91 N in comparison with the control group (11.76±1 N).

**Conclusion:**

This *in-vitro *study demonstrated that the retention of O-rings was affected when soaked in cleansing solutions. NaOCl caused more reduction in retentive value compared to effervescent cleansers and would not be recommended for cleansing O-rings. These results should be interpreted clinically and the role of other factors in the retention of O-rings should be considered in order to recommend the best cleanser for O-ring overdentures.

## Introduction

Mandibular edentulous patients require more denture stability and improvements in mastication. Mandibular overdentures can satisfy the needs of these patients much better than conventional full dentures.[1] Overdentures are less expensive than implant-supported fixed prosthesis and are more popular among edentulous patients. Problems associated with mandibular dentures such as little retention and stability, reduced function, disturbances in speech, sensitivity and irritation of soft tissues has led to the recommendation of implant-retained overdentures as the treatment of choice for these patients.[2-5]

Overdenture wearers must follow specific homecare instructions to clean their dentures and maintain oral mucosal health.[6-9] Chemical solutions for denture cleansing should be used because they are more effective than mechanical methods.[6, 10-11] However, chemical solutions may have deleterious effects such as bleaching the acrylic resin, metal corrosion and transient or permanent destruction of soft liners.[6, 12]

Overdentures use different retentive systems such as O-rings, locator attachments and Hader clips. Only a few investigations have studied the effects of denture cleansing solutions on the retention of these attachments. You *et al.* and Nguyen *et al.* evaluated pink locator attachments[13-14] and in a study by Varghese *et al.*, yellow Hader clips have been studied.[15] To the best of our knowledge, no research on the popular O-ring attachments has been performed. Therefore, this study was conducted to evaluate the effects of some common denture cleansing solutions on the retention of Dio orange O-rings of the DIO system, as one of the chair side O-rings. The null hypothesis of our study was that denture cleansing agents would not affect the retention of Dio orange O-rings.

## Materials and Method

This research adopted similar methods to those used previously in Varghese *et al.*[15] In this experimental* in-vitro* analysis, we fabricated two metal flasks and tested forty O-rings for a six-month period.

Fabrication of the metal flasks with implant lab analogues and O-ring housings

**Figure 1:**
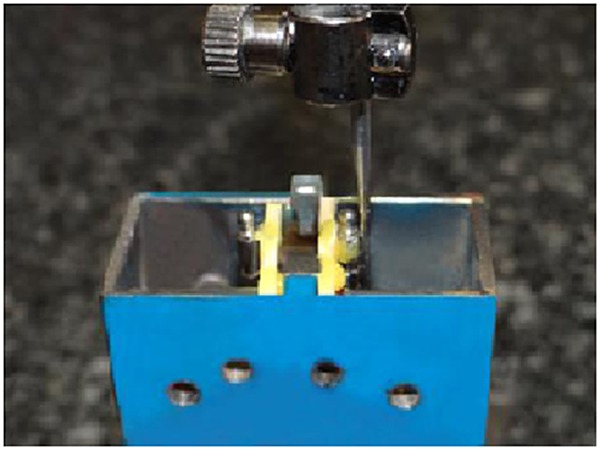
Checking the ball abutment implant analogues with surveyor to ensure their parallelism , the analogues were maintained in their place during acrylic packing using wooden struts and sticky wax.

Initially we constructed two metal flasks made of galvanized iron, with dimensions of 2.5 × 2.5 × 5 cm. One flask was for placement of the implants, whereas the other was for the O-ring housings. A notch with a dimension of 4×4×4 cm was cut out on one of the flasks. The other flask had a complementary raised area of the same dimensions as the notch, which enabled us to ensure that the flasks would always be seated in the same position. A number of holes were cut into the flask walls to allow the acrylic resin to penetrate during packing, ensuring a tight junction with the metal flasks. Two metal ridges were located on the outer upper and lower surfaces of each flask that enabled us to hold and place the flasks into the Zwick/Roell Z020 testing machine (Zwick GmbH & Co. KG; Germany). Two ball abutment implant analogues (SABA 3510; DIO Implant System, Korea) were placed in one of the flasks at a distance of 1 cm from each other and checked with a surveyor (Ney Dental Surveyor; Dentsply, Ballaigues, Switzerland) to ensure their parallelism (Figure 1).

**Figure 2:**
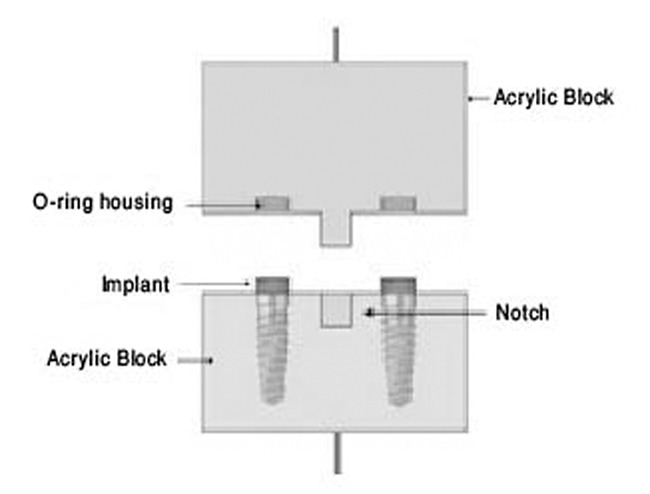
The acrylic blocks used for testing O-ring attachments. The implant analogues were placed in lower flask, and the O-ring housings were placed in the upper one.[14]

Next, the flasks were filled with cold cured Acropars acrylic resin (Marlik Company of Medical Industry, Iran). In this manner, the top of each implant was 1 to 2 mm above the acrylic surface. After completion of the acrylic resin setting, we placed the housings (DBPM 201; Dalbo Plus, DIO Implant System, Korea) of the O-rings (OR 0450O; O-ring, Dio Implant System, Korea) on top of the implant analogues. In order to preclude acrylic adhesion of the two blocks, one layer of separating medium covered by Vaseline was placed on the first acrylic surface. Subsequently, we packed the second flask with the same acrylic resin and the first flask was placed upside down on the second flask such that excessive acrylic resin could be extruded. After the acrylic resin cured we separated the flasks. The O-ring housings were embedded into the second acrylic flask (Figure 2).

**Table 1:** Experimental design and soaking periods.[15]

**** **Denture cleansing solution**	**Immersion time based on manufacturers’ instructions**	**Total immersion time** **(equivalent for six months)**
Control group	-------	1440 h
Corega Tabs	15 min	45 h
Professional Tabs	3 min	9 h
NaOCl	15 min	45 h

*Testing procedure*

The O-rings were divided into four groups .The groups were soaked in either NaOCl diluted 1:10 in tap water; Corega Tabs (Corega Denture Cleanser Tabs, Ireland); Professional Tabs (Professional Denture Cleanser Tabs, Switzerland); or water (control group). The O-rings of each group were placed into perforated plastic bags. A small marble in a small plastic bag was placed in each bag of O-rings. In this way, we ensured that the perforated bags would be immersed in the solution during the entire soaking period. The bags were immersed in each solution according to the manufacturers’ instructions for the time equivalent of six months.[15] For example, Corega Tabs and NaOCl solutions were changed every 15 minutes, whereas Professional Tabs solution was changed every three minutes (Table 1). Prior to changing the solution, the plastic bags that contained the O-rings inside were rinsed with tap water for 15 seconds.

After equivalent time for six months, one operator inserted the O-rings into their housings within the acrylic block after which the flasks were placed together, ensuring that the ball attachments were placed in the O-rings. Peak load-to-dislodgement was measured and recorded by a testing machine (Zwick/Roell Z020). The tensile force applied was 2 inch/ minutes, because it has been proven that patients would remove their dentures at approximately that speed.[16]

Data recorded by the testing machine were imported to SPSS version 18 software and analyzed by one-way ANOVA and Tukey's HSD tests. *p*≤ 0.05 was considered as significant.

## Results

**Figure 3:**
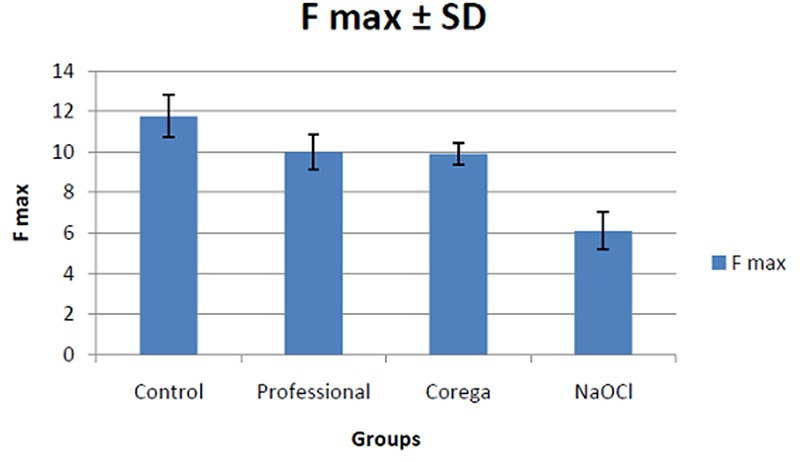
Comparison of mean of maximum force (F max) required for dislodging the two metal flasks among the groups.

The peak load-to-dislodgement that was recorded by the testing machine was significantly different between control group and the other 3 groups (Tables 2). Maximum force required for dislodging the two metal flasks was 11.76±1 N within the control group, and 6.1±0.9 N in NaOCl group (Figure 3).

Data analysis, based on One-Way ANOVA test, indicated significant difference between control groups and the other 3 groups (*p*≤ 0.001). Data from all 4 groups were also mutually analyzed with Tukey HSD test. Statistically significant difference existed between all groups, except between Corega tabs and Professional tabs.

**Table 2:** Multiple comparison results of groups tested. Mean of maximum force (F max) required for dislodging the two metal flasks in testing groups.

	**Fmax (Mean±SD)**	**P Value Ɨ**	Multiple comparison results*
1.Control group	11.67±1.03*a	<0.001	1 vs 2(<0.001) 1 vs 3(<0.001) 1 vs 4(<0.001)
2.Corega Tabs	9.99±0.86*b	<0.001	2 vs 3(0.997) 2 vs 4(<0.001)
3.Professional Tabs	9.91±0.54*b	<0.001	3 vs 4(<0.001)
4.NaOCl	6.11±0.91*c	<0.001	

Ɨ: using one-way ANOVA

*: using Tukey test

Different letters show a significant difference between groups based on Tukey test.

## Discussion

Overdentures can be retained by clips, attachments, or magnets.[17-20] One of the most common methods for retaining implant overdentures is using a ball shape implant and an O-ring. This in-vitro study investigated the effect of denture cleansing solutions on the retention of Dio orange O-rings of the DIO system as a clinically popular attachment. Although the cleansers used in this study have different chemical compositions, they are all oxygenating agents, they are widely available in market and used by lots of overdenture patients. Our results indicated that NaOCl, Corega Tabs, and Professional Tabs lead to reduced retention of Dio orange O-rings (*p*≤ 0.05). This decrease in retention was clinically important for NaOCl (48%). The observed decrease was approximately15% for Corega and Professional Tabs. There was no statistically significant difference in reduced retention in the Corega Tab group compared to the Professional Tab group.

Considering the study results, it seems that the null hypothesis of this research has been rejected, as we have proven that chemical cleansing solutions affect O-ring retention.

In contrast to our results, Varghese *et al.* have evaluated Hader clips and concluded that daily 15-minutes use of NaOCl caused increased retention. They tested seven groups (each with 18 yellow Hader clips) with different cleansers. Their results also showed that daily use of NaOCl and effervescent cleaning agents such as Polident and Efferdent for eight hours did not affect retention of the Hader clips.[15] Which could be attributed to the difference in O-ring composition and its design characteristics compared to the Hader clip.

Nguyen *et al.* have shown that while Polident and Efferdent cleansing agents slightly decrease retention; the use of NaOCl for eight hours per day lead to significant reduction in retention of locator attachments (82.7%).[14] These results agreed with the results of the current study. In their study, Listerine increased retention. The results for the latter three cleansers might be clinically unimportant.[14]

Recently, You *et al.* have evaluated the effects of denture cleansing solutions on the retention of pink locator attachments after multiple pulls. This research has better simulated clinical conditions, and have tested the samples during 548 cycles of insertions and removals. As a result, NaOCl significantly decreased the retentive value of locators, whereas Listerine significantly increased the retention of the locator attachments. These results have agreed those of Nguyen *et al.* and the current study.[14] However, Listerine is recommend as a denture cleanser and further studies evaluating its effects should be conducted.

Although we have evaluated the effects of cleansing agents on the retention of Dio orange O-rings, we expect that these solutions similarly affect other O-rings since different colored O-rings that are associated with different implant systems have similar polymeric compositions.

This study had several limitations. First, the O-rings were continuously soaked in the cleansers for a simulated period of six months; however more changes might appear after a longer period of time. The continuous soaking of O-rings is actually different from the clinical situation where periods of soaking are interrupted with periods of use, as patients wear their dentures during the day and soak them in denture cleansing solutions at night. Etimovska E., *et al.* showed that retentive values of attachments tested in their study were significantly reduced over time after multiple pulls,[21] however, we did not considered this situation in our study. We also did not consider the thermal and chemical conditions of the oral cavity which might have deleterious effects on the O-rings. Therefore, further researches considering thermocycling and multiple pulls with longer testing times in this context are indicated.

## Conclusion

The finding of this *in-vitro* study shows that O-rings are affected by denture cleansing solutions. NaOCl leads to more decrease in O-ring retention compared with effervescent cleansers. Hence, NaOCl is not recommended as a cleansing solution for patients who use overdentures with O-rings. To recommend the best cleansing agent for overdenture users, the *in-vitro* verdicts of this study should be confirmed clinically and the role of other factors in the retention of overdentures should be considered.
